# EndoNet: an information resource about the intercellular signaling network

**DOI:** 10.1186/1752-0509-8-49

**Published:** 2014-04-24

**Authors:** Jürgen Dönitz, Edgar Wingender

**Affiliations:** 1 Institute of Bioinformatics, University Medical Center Göttingen, Goldschmidtstr. 1, 37077 Göttingen, Germany

**Keywords:** Intercellular network, Endocrine network, Signaling, Hormone, Receptor, Systems biology

## Abstract

**Background:**

In multicellular organisms, an intercellular signaling network communicates information from the environment or distant tissues to defined target cells. Intercellular signaling (mostly mediated by hormones) can affect the metabolic state and the gene expression program of target cells, thereby coordinating development, homeostasis of the organism and its reactions to external stimuli. Knowledge of the components of the intercellular signaling (specifically: the endocrine) network and their relations is an important, though so far a largely neglected part of systems biology.

**Description:**

EndoNet is an information resource about the endocrine system in human. The content of this database comprises information about the biological components of the endocrine system, like hormones, receptors and cells, as well as their relations like the secretion or the binding of a hormone to its receptor. All data within EndoNet have been manually annotated from the scientific literature. The web interface of EndoNet provides the content by a detailed page for each component. These pages list information about the component, links to external resources including literature as well as to related entities of EndoNet. The anatomical ontology Cytomer is used, in conjunction with the Ontology Based Answers service (OBA), to query and list related anatomical structures ranging from the level of individual cells to complete organs. While querying the web interface the user can add components to an individual network. This network, or the complete network stored in the database, can be further analyzed in a configurable pipeline or can be exported in various formats.

**Conclusion:**

EndoNet is an important and unique information resource about the intercellular signaling network. Since the intercellular network is an integral part of systems biology, EndoNet provides essential information for analyzing interaction between different cellular networks.

## Background

A multicellular organism faces the challenge to coordinate the activities of different cell types, tissues and organs. Since not every cell is in immediate contact with all those it has to communicate with, the transport of signals to the right target requires a complex system. Signaling is not limited to the internal communication of an organism, but also transmits external stimuli so that the organism can react to its environment. A century ago, 1905, the study of the endocrine system started with the definition of hormones by Ernest Starling as messengers that are secreted by endocrine glands [1]. Today, with a more systems biology-driven view of complex organisms, this early definition has widened to cover all secreted messengers involved in the intercellular communication. Molecular studies disclose details of the endocrine system, like the expression of a receptor in a specific tissue, the binding of a hormone to a receptor, or the molecular effect of a receptor’s activation. This kind of detailed information is scattered throughout the primary literature and reviews. Clinical studies describe the impact of factors from the environment or administered substances on phenotypes. Also there is usually a molecular pathway between the external stimulus and the observed phenotype. In many cases, these pathways have not been demonstrated at the molecular level, rather the components involved have been inferred from the observations made, combined with pre-existing knowledge.

While a huge amount of detail is known about the individual components of the endocrine networks, only a handful of pathways, called endocrine axes, have been characterized; each axis consists of a number of steps, i.e. subsequent hormonal induction or hormone secretion events. Also compared with the intracellular network, very little is known about the inter-cellular signal flow along such endocrine axes. Examples are the hypothalamic-pituitary-gonadal axis [2], the fight-or-flight response [3] or the hypothalamic-pituitary-thyroid axis [4].

Several data resources like Ensemble [5], RefSeq [6] or Gene Ontology [7] store information about genes and gene products. The high-throughput measurement of gene expression levels, protein-DNA interactions or protein-protein interactions has become common practice. Based on these data regulatory and metabolic networks have been reconstructed and are available from databases like Reactome [8], TRANSPATH [9] or KEGG (Kyoto Encyclopedia of Genes and Genomes) [10]. All these resources and methods focus on intracellular pathways, while the intercellular network is largely ignored. Even text books of systems biology [11] or reviews dealing with the impact of the environment on the genetic network [12] usually start the network analysis with the activation of a receptor or a phenotype considering the signal flow to the receptor as black box.

EndoNet aims to fill this gap by building up an information resource about the signal flow outside of the cell boundaries When EndoNet went online in 2005, it was the first database focusing on the intercellular network and constantly evolved since then [13,14]. To the best of our knowledge, only one other database exists that focuses on hormones and receptors but this puts less emphasis on the underlying network (HMRbase, status 2009 [15]). Due to the importance of the endocrine network for systems biology, more attention needs to be paid to the intercellular network. In addition to the continued annotation of EndoNet since 2008 [14], the data model was extended to include entities for phenotypes and external stimuli. A new web interface has been developed, which provides access to the data from different angles and includes a better support for browsing and data analysis. The anatomical ontology Cytomer [16,17] has been integrated into the detail pages and the search function of this web interface, in order to manage the diversity of anatomical structures above the cellular level.

## Construction and content

### The content of EndoNet

The scope of EndoNet is the intercellular network in humans. The endocrine system with the classical hormones secreted by a limited set of glands is well known and is the core of the intercellular network. An increasing number of components in the database are not classical hormones like growth factors or cytokines. In EndoNet, no distinction is made between classical hormones and other messengers, both terms are used interchangeably. If a messenger is secreted in a controlled way by its donor cell and has a specific target, the messenger is within the scope of EndoNet. The involved cells, messengers and receptors should be human, or the scope of the experiment should be the human intercellular network when a model organism is used.

**Figure 1:**
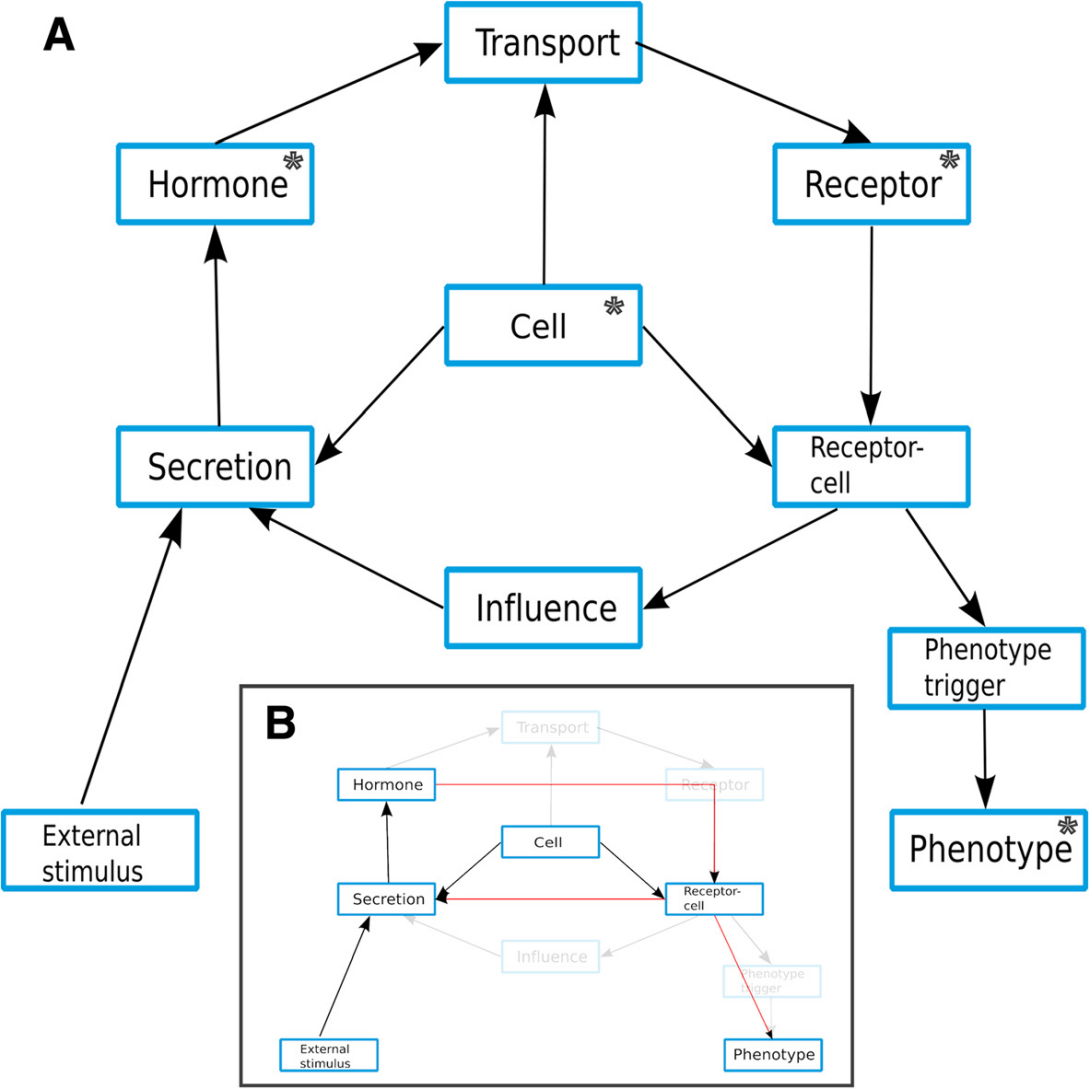
**Overview of the EndoNet structure. A:** Shown are the major entities of the database and their connections. In this abstract outline, all attributes and entities that are only needed for database modeling, like the subunits of the hormones and receptors, are omitted. Also the entities referring to the literature sources and comments are left out. Each shown entity has a commented connection to literature. The entities which are marked with an asterisk can be searched by their name in the web interface. All entities can be retrieved by their ID. **B:** Transformation of the complete network to the signal flow network. The opaque entities and relations are removed and their information has been moved to the neighboring entities. The red relations are inserted for modeling the signal flow network.

The network concept of EndoNet reflects the fact that many messengers mediate signals in a cascaded manner, where one hormone triggers synthesis and/or secretion of the next one, turning the target cell of the first hormone to the source cell of the following messenger. At the bottom end of such a cascade, a target cell is triggered to adopt a certain phenotype. The entities of EndoNet are the components involved in the intercellular signal flow and are outlined, together with their relations, in Figure 1A. This model is the representation of the network stored in EndoNet and also the foundation of the database model which is completed with the required linking tables. The first one is the hormone or, in more general terms, the messenger. The target of a messenger may be one or several receptors. Most often, the receptor for an intercellular messenger is a transmembrane receptor with the cell membrane as border between inter- and intracellular pathways. However, nuclear receptors are also considered as targets of intercellular messengers if these can pass the cell membrane like the members of the steroid hormone family. The cell itself is handled as black box in EndoNet. The intracellular pathways are subject to specialized databases like TRANSFAC, Reactome or KEGG and the components of EndoNet are linked to these databases. In EndoNet, two kinds of effects are considered that are elicited by the activation of a receptor: (a) the influence on the secretion of another hormone by the target cell, or (b) triggering of a certain phenotype. Either case is represented by a single connecting entity from the receptor to the next secretion event or from the receptor to the phenotype. This link summarizes all intracellular reactions and connects the receptor with the outcome of its activation. The sources of messengers are the cells that secrets them, and their targets are cells that express the corresponding receptors.

The described biological entities are complemented by intangible ones like phenotype or linking entities like the cell-receptor-combination. There are many-to-many relations that are modeled as entities comprising further attributes and references to the literature. These are:

–the secretion events, which are connecting the source cell and the hormone,

–the connections of the messengers to the corresponding receptors, modeled by a node which links the literature of the binding event and the corresponding transport medium (such as blood or lymph), and

–the expression of a receptor in a given cell-type (receptor-cell combination).

The data in EndoNet have been manually annotated from the scientific literature, that is primary research papers and some text books [18-20]. The annotation has been focused on specific topics, like lipids as messengers or lipid metabolism, or has aimed to complete the network. Some kind of information is easier to find in the literature than other. Publications about the expression of a receptor in specific tissues is found quite easily while the intercellular links between a receptor and the next secretion event is mentioned less often and not so clear. Therefore, a second focus of the annotation is to add the missing edges in the network. The source of the annotation is linked to the components of EndoNet. In most cases, a sentence summarizes the information which has been extracted from the publication. In addition, these links to the literature have been added to those entities, which mainly connect other entities like the receptor cell combination. In this way, publications describing the tissue specific expression of a receptor are not cumulated by the receptor entity.

### Use of the Cytomer ontology

Although the annotation policy of EndoNet is to gather information as precise as possible, the available data in literature is often at the level of tissues, organs or even larger structures. It may happen that the searched information is on another level, e. g. on the level of cells when an organ was searched or vice versa. To address this issue all anatomical structures available in EndoNet have been linked to the anatomical ontology Cytomer [17]. The ontology sets the cells, tissues and organs in relation and is used by the EndoNet web interface to assist the user in finding the appropriate information. During the search for each anatomical structure that fits the search pattern, all those substructures are listed for which additional information is available in EndoNet. Applying the same logic substructures and higher level structures are included on the detail pages. To retrieve the relevant information from the ontology the Ontology Based Answers service (OBA service) [21] is used. The OBA service provides access to ontologies, in this case to Cytomer. It also implements knowledge about specific ontologies, like the meaning of the used relationships, to provide functions that are specific for a given ontology. For EndoNet we use the OBA service to find related anatomical structures. Therefore the anatomical entities of EndoNet are annotated with the corresponding ontology ID from Cytomer. To limit the results to those entries for which information is available in EndoNet a list of anatomical entities is uploaded to the OBA service in advance. Using this filter avoids an overload of the EndoNet page with information from the ontology unrelated to EndoNet. Cytomer contains by far more entities than EndoNet makes use of (around 10,000 ontology classes as compared to 416 anatomical structures in EndoNet).

### Network transformation

The described network reflects the annotated data with the highest detail level available. Depending on the problem addressed when working with EndoNet, this network may have to be converted. If the focus is put on the flow of a signal through the intercellular network, a simplified graph may be most suitable. Linking entities can be omitted by linking the neighboring entities directly and copying the additional attribute to the edge. The result of the transformation of EndoNet’s network to the signal flow network can be seen in Figure 1B. The transport node always has one incoming (from the hormone) and one outgoing (to the receptor) edge, but has also assigned comments and references to literature and to the involved transport medium. This supplementary information is not needed for the graph representation of the endocrine network and therefore also the node can be removed. This is generally achieved by the following procedure for the sequence of the tree nodes A, B and C.

–Link node A directly to node C

–Copy relevant information from node B to the edge which was created in the previous step.

–Remove node B

For the previous example the entities for the nodes A, B and C are the messenger, the transport node and the cell-receptor combination. The copied information is the transport medium. A similar situation represents the influence node which connects the receptor-cell combination with the secretion. The type of influence, i. e. negative or positive effect, can be copied to a newly created edge between cell-receptor combination and the secretion. Afterwards the influence node can be removed without loss of information.

The last transformation during construction of the signal flow network is the removal of the phenotype trigger. This many-to-many relation is represented in the graph by the edges between receptor-cell combinations and phenotypes.

## Utility

The web interface of EndoNet provides different views of the data. On a basic level detail pages for each component present all available information (see Detail pages below). Moreover, a simple path analysis can be performed on the web interface like finding the paths between two nodes in the intercellular signaling network. A configurable processing pipeline provides additional methods to analyze the network. The web interface also supports the export of networks in various formats to provide the option of further studies.

### Search interface

EndoNet’s search interface allows the user to retrieve intercellular network components by their name, IDs or synonyms. The search results are grouped by the main biological entity types: messengers (hormones), receptors, anatomical structures and phenotypes. For each anatomical structure also the corresponding substructures are listed about which information is available in EndoNet.

### Detail pages

**Figure 2:**
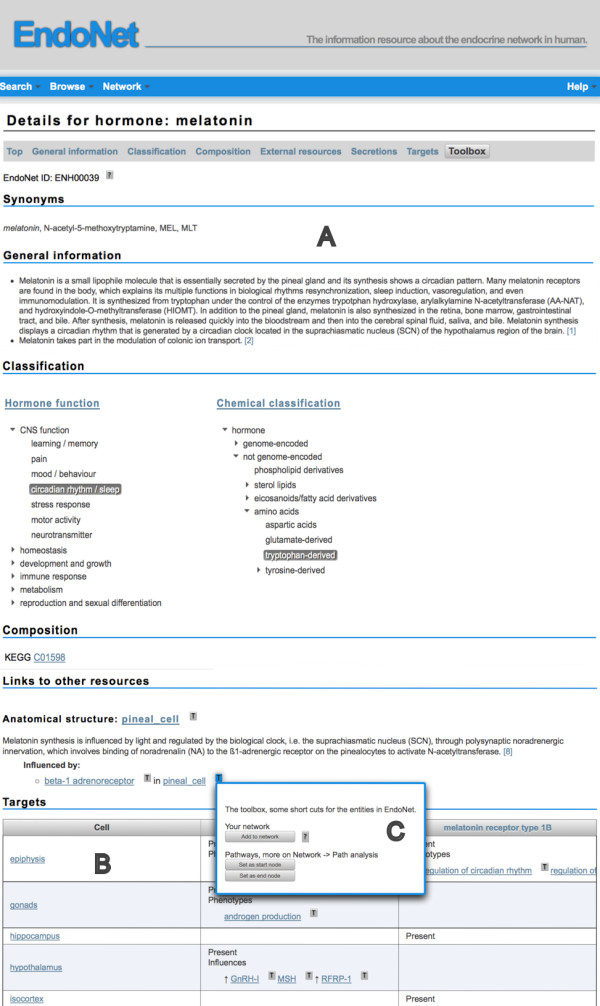
**Detail page for hormone entries.** Shown is the detail page for the hormone melatonin. The detail pages for the other entity types of EndoNet follow the same structure, which starts with general information about the component followed by external link and links to related components of EndoNet **(A)**. The related components are not restricted to the direct neighbors but include the information the user might be interested in. In case of a hormone this also includes the target receptor, the target cell and the tissue specific phenotype or influence on the next secretion event (table marked with **B**). Next to each EndoNet component a button allows to open a toolbox (marked with **C** in the Figure). This toolbox provides options to add a component to a user defined network or to select it for the path analysis without the need to go to the corresponding component’s detail page.

For each entity of EndoNet the available information is provided on a detail page. In Figure 2 an example page for a messenger is given. The pages are divided into several segments, which are kept as consistent as possible for the different entity types. Each page starts with general information about the displayed component like synonyms, general comments and links to other resources (Figure 2A). For anatomical structures a list of larger structures and substructures is included, similar to the search results.

Beneath the information about the displayed component itself the connected components are listed. These may be the direct neighbors or other relevant components. For example, in case of a hormone the user can find information on the upstream secretion by the source cell(s) and also about the upstream receptor that triggers the secretion in response to another hormone. For the linking entities, in this case secretion and influence, the comments and the referenced literature are included. In case of the target of a hormone all this relevant information is organized in a two dimensional table with the target receptors and target cells on the axes and the tissue-specific effects (influence or phenotype) in the table cells (Figure 2B). Next to each component is a button to open a toolbox. With this toolbox the user can add the component to a customized network or mark it for a path analysis (Figure 2C).

### Path analysis

**Figure 3:**
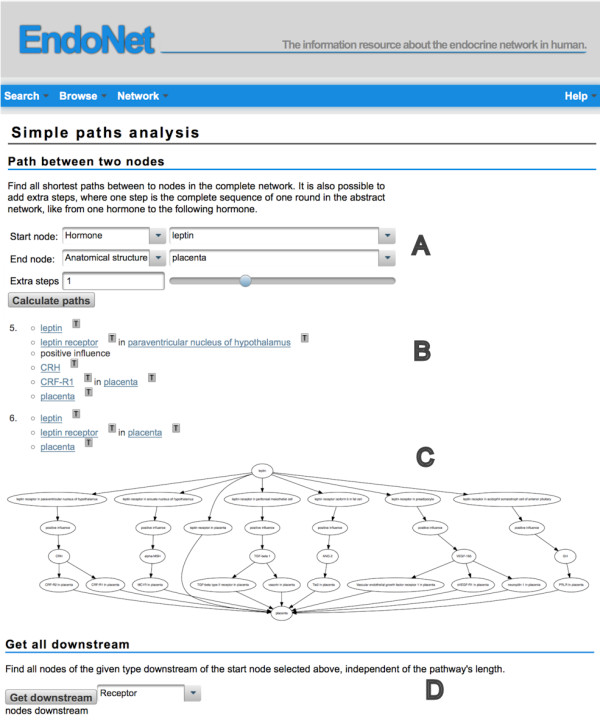
**Network analysis page.** On the screenshot the steps are marked that are needed to perform a path analysis between two nodes of the virtual network. First the entity type and the entity for the start and end node has to be selected **(A)**. Optionally a number of extra steps may be selected to get not only the shortest path but also those pathways which are longer than the shortest path by the specified number of steps. One step is interpreted as one round in the abstract network, e.g. from hormone to the next hormone. The result of the pathway search is a list of pathways between the two selected nodes **(B)**. In the list of pathway steps some entity types of the abstract network are omitted for better readability. The information of these steps is aggregated with the neighboring steps. The graph below of the pathways summarizes all listed pathways to show potential common steps of the pathways **(C)**. Beside the paths between two distinct nodes it is also possible to show all nodes of a given type downstream of a node **(D)**.

Following the links on the detail pages the user can migrate from one component of the endocrine network to the neighboring ones. A more comfortable method to follow longer paths and of finding out upstream/downstream relations of the components is provided with the path analysis option on the EndoNet web interface. The corresponding page is captured in Figure 3.

To find paths between two nodes they should be selected as start and end node, either through the toolbox or on the analysis page (Figure 3A). The algorithm performs a breadth-first search on the complete network. The result is a list of all shortest paths between the two given nodes. In the pathways the steps and their sequence are the same as in the abstract network with the exception of the secretion or transport events and receptors. These steps are aggregated with the neighboring components without loss of information. If a hormone is listed below a cell or organ the reader intuitively deduces that the hormone is secreted from this anatomical structure. (Figure 3B). Below the list with the paths, a network comprises all paths in a single figure (Figure 3C).

The shortest possible path in an artificial network may not be the one observed in vivo. Hence, the path analysis in EndoNet is not restricted to the shortest paths, but a number of extra steps can be specified. A step is here interpreted as one round in the abstract network, e.g. from one hormone to the subsequent one.

A variant of the path analysis is a downstream search from a defined start node to all components of a given type. Such a search is suitable to get those components of the endocrine system that might be under the control of an upstream regulator. An example for such a search would be to retrieve all receptors that are found in downstream pathways starting with leptin, independent of the path length. The result could then be the starting point for an intracellular network analysis with the receptor(s) as connection points to reveal the influence of a single signal to the intercellular network (Figure 3D shows the start option, results are not displayed).

### Annotated pathways

Complementary to the computationally composed pathways, a number of manually annotated pathways are provided. In endocrinology some well-known hormone axes exist like the hypothalamic pituitary growth hormone axis or the fight-or-flight response. In EndoNet such pathways have been annotated as a sequence of linked pathway steps. In EndoNet the components of these pathways are preferably represented at a more abstract level, organs are favored to cells and clinical phenotypes to molecular ones. The visualization of the pathways is abstract, too. With the help of the pathway graphs the information flow can be easily traced.

### Processing of the network

The web interface provides the possibility to define and process own networks composed from components on EndoNet. On the “Process network” page, this compiled network can be revised, processed and exported.

**Figure 4:**
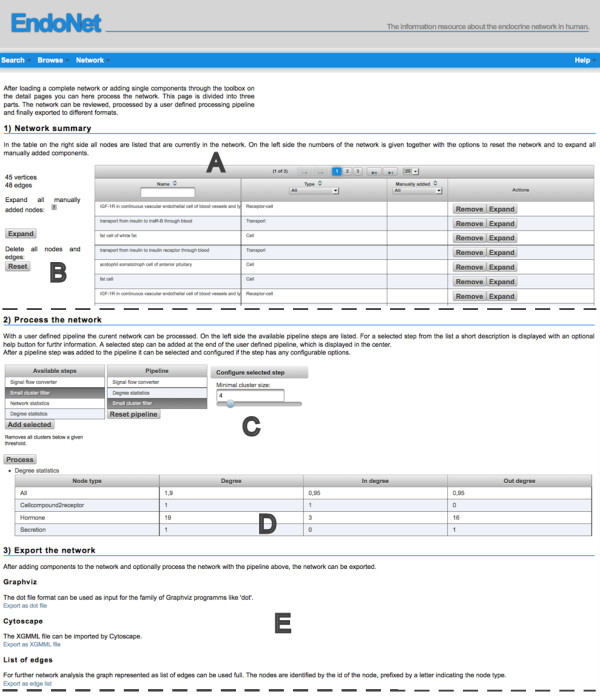
**Network processing page.** On this page the user-defined network can be revised, processed and exported. All components the user has added to the network are listed in the table on the top of the page **(A)**. Individual entities can be expanded or removed. All manually added components can be selected with the button next to the table **(B)**. The revised network can be processed with a configurable pipeline **(C)**. The pipeline step can filter the network (e.g. immune system filter), process the network (e.g. signal flow converter) or print information about the network (degree statistics) **(D)**. Finally the network can be exported into various formats **(E)**.

All components the user has added to the network are listed in the table on top of the page (Figure 4A). The table’s data can be sorted and filtered and single components can be removed or expanded. The expand button next to the table will expand all components that were manually added by the user (Figure 4B). Which nodes are added during the expansions depends on the type of the expanded node. The aim of this step is to include only the related nodes to the network without adding large parts of the network. This would happen if all reachable nodes would be added to the network. On the help page for each node type an abstract network is given, with the nodes marked which will be considered during the expansion. The selection of nodes to be included during expansion of the network has to be limited to the most relevant related nodes without missing important information. Otherwise the number of nodes would rise quickly, due to the density of the network.

User defined networks, which are created on the EndoNet webpage follow the semantics of the abstract network described above. This network may be filtered or converted in the next step on the processing page (Figure 4C). A set of filters are available which the user can select and add to a processing pipeline. Examples are the signal flow converter, the immune system filter and network statistic pipeline step. The signal flow converter transforms the network to a layout concentrating on the flow of signals in the intercellular network like described above. The “immune system filter” and the “small cluster filter” filter nodes from the network. The immune system is an important part of the intercellular network. However, if the focus of a study lays on a classical endocrine topic, like the growth factors, the components of the immune system can be excluded. Sources and targets of growth factors will be also involved in reactions of the immune system, but only under certain circumstances. The immune system filter uses the annotated function of the hormones as starting point. The hormone function is annotated from literature and displayed on the detail page of the messenger. The filter stepwise removes the following nodes: 1) Hormones that have “immune system” or any subclass assigned as annotated function. 2) Receptors that bind no other messengers than those filtered in (1). 3) Remove the influence and phenotype trigger nodes downstream of the deleted receptors. 4) Remove the secretion events for the hormones deleted in the first step.

Orphan nodes and small clusters that can occur, for example after the application of the “immune system filter” can be removed with the “small cluster filter”. The “small cluster” filter removes all clusters from the network with a size below a threshold that can be set by the user (four by default). A pipeline step can also output information about the network, like the number of each instances of a node type. Such a step may be also applied multiple times to the pipeline, i.e. before and after the immune system filter (Figure 4D).

The last option on the network processing page is the export of the user defined and optionally pre-processed network (Figure 4D). Various file formats are available for the export, so that a further analysis of the network with local tools can be easily achieved. The exported nodes of the network consist of the EndoNet ID (edge list, Pajek format) or contain them. These IDs can be entered in the search field of the web page or can be appended to the URL of the search page to retrieve information that is not available in the export format. A more detailed description of the pattern of the IDs and the URLs is available on the help page. The most flexible export format is the XGMML format; these files contain also additional information, like the name of the component or the assigned hormone function classes.

### Statistics

Table 1 summarizes the number of entities of the main types of the EndoNet database. For comparison, the numbers from 2007 are given [14]. The main increase was achieved by enhancing the size of the underlying graph, i.e. by increasing the number of edges. Thus, we focused on adding intra- and intercellular links (increased by 103% and 27%, resp.), receptor-cell combinations (49%), which are the effective targets of hormonal actions, and with newly introduced entities such as phenotypes and external stimuli. Besides, considerable more evidences were added, which led to a 59% increase in the number of references, and 34% more explanatory free-text comments support the user’s understanding of the formalized representation of literature data. We also put efforts in increasing the number of manually annotated hormonal pathways to a total of 9 now, which is a particularly time-consuming task. The number of external stimuli will rise when the data model for this entity is finished.

**Table 1:** Statistics of the EndoNet database

**Entity type**	**Number (3. 2014)**	**Number (9. 2007)**
Messengers (Hormones)	740	637
Cells/tissues/organs	416	314
Phenotypes	533	-
Intracellular links (influence)	521	257
Intercellular links (hormone binding)	1095	861
Secretion events	2252	1920
Receptors	615	500
Receptor cell combinations (target)	2323	1555
External stimuli	23	-
Annotated pathways	9	6
Publications referenced	3060	1926
Comments	6818	5071

## Methods

The web interface is implemented as JSF (JavaServer Faces) utilizing the PrimeFaces component library. The data are stored in a MySQL database and fetched with Hibernate as JPA (Java Persistence API) implementation. The search for components is implemented with simple queries like “SELECT DISTINCT h FROM Hormone h LEFT JOIN h.names AS n WHERE lower(n.name) like :pattern”. The properties of the retrieved component and the related entities are loaded by JPA.

For the communication with the OBA service the OBA client is added to the class path and uses the REST protocol to contact the server. The anatomical entities stored in EndoNet have corresponding Cytomer ID annotated. After an update of EndoNet’s data the set of ontology classes used in the database is transferred to the OBA service. For the search of related anatomical structures the functions “findUpstreamInSet” and “findDownstreamInSet” in conjunction with the previous uploaded set.

The graphical representation of the pathways is done with the help of the Graphviz. The nodes and edges of the network are streamed in the dot file format to the dot file of the Graphviz suit. The resulting scalable vector graphic (SVG) is added to the webpage and rendered by the browser.

## Discussion

The data stored in EndoNet is annotated manually from original publications as collection of, in a first step unrelated, molecular events. The information available in the literature is not equally distributed over the molecular steps of the signal flow. While, for example, the expression of a receptor in a tissue, the receptor-cell combination, can easily be found in the literature, reliable information for the downstream effect of the receptor’s activation, the phenotype or influence of the next secretion, is hard to find. Hence, this type of contents has already been put in the focus of the EndoNet annotation and will be expanded in the future. In the past the amount of data in the database was significantly increased [14] and further annotation will expand the network and add more details. Compared to intracellular networks with thousands of genes or proteins, the intercellular network of EndoNet is smaller in numbers. However, the network is very dense and contains a large amount of crosslinks between the entities. The vast majority of EndoNet’s entities belong to the same network and the majority of the nodes can be reached from any other node. The remaining few small disconnected sub- networks will be linked to the main network, while the annotation goes on. Due to the non-hierarchical network already small subparts of the graph are hard to visualize because the network will not fit to a tree-like or hierarchical layout. On the EndoNet’s web interface graphical representations are limited to the manually annotated pathways and the path analysis of at most 20 pathways. A more convenient tool for an interactive visualization and exploration is, for example, Cytoscape [22] which also supports the EndoNet export format XGMML. An HTML5 based viewer, like Cytoscape Web, could be a solution for an interactive and integrated viewer for the next release.

The external stimulus is a natural starting point for endocrine pathways. They are already included in the model of EndoNet and annotation started, mainly for known endocrine axes. But still external stimuli are underrepresented in EndoNet. The endocrine system has to react to a wide range of stimuli like light, stress or a change in the physiological state. The data model should not only incorporate the different nature of the diverse stimuli but also allow a dynamic simulation of the triggered pathways. For example the time scale ranges from seconds (adrenalin) to years (growing up), or may have a pulsatile component, again in different time scales (day-night rhythm, monthly cycle). At the moment such data types and parameters are collected as input for a modeling of external stimuli in EndoNet.

Each single step of EndoNet’s network corresponds to experimental findings documented in literature. The sum of these steps is a theoretical network of all interactions of the endocrine system comprising different circumstances like age, sex or metabolic state of the organism. Algorithms and methods have to be developed to filter out a sequence of molecular events that will occur in vivo under given circumstances. The path analysis on the EndoNet webpage outputs all shortest paths between two nodes independent of any circumstances, whereas the manually annotated pathways (mainly endocrine axes) are limited in number and restricted to already known pathways. Algorithms and methods are needed to extract these pathways from the reference network that occur in vivo under given circumstances.

The downstream search on the “Path analysis” page provides the option to analyze the impact of the endocrine network to the cellular ones. If receptors or phenotypes are selected as target for a downstream search they can be used as handover points between these two domains. The BioUML workbench [23,24] demonstrates this already by an integrated analysis of both networks, with the receptors as interconnection points. Another useful intersection of EndoNet with other networks is the use of phenotypes. The majority of phenotypes in EndoNet is of molecular or cellular nature like cell proliferation and is linked to GO terms. They can be utilized as common input for a wide range of analysis methods. EndoNet handles the cells as a black box and connects the input to a cell with the outcome with one single link. This link can be used to limit the intracellular pathways downstream of a receptor or to enhance the methods to predict such pathways using the known outcome as validation. More abstract phenotypes from clinical studies are linked to data resources like Medline Plus (http://www.nlm.nih.gov/medlineplus), OMIM (Online Mendelian Inheritance in Man, http://omim.org) or the Diseases database (http://www.diseasesdatabase.com). Systems biology aims to provide an integrated, holistic view of a biological system for a comprehensive understanding. If the system is a multicellular organism, it is mandatory to understand and model the communication between the cells, which orchestrates their activities and responses to environmental stimuli. EndoNet adds the intercellular signaling network and provides multiple interaction points to other information resources. With knowledge of the intercellular network the (downstream) effect and (upstream) reasons for changes in the metabolic or gene regulation network can be taken into consideration. For clinical studies, models of the intercellular network can provide mechanistic explanations of the effects downstream of an external stimulus, such that its effect on the whole organism can be explained and maybe even predicted. Thus, any systems biological study of cellular responses in the context of a tissue or a whole organism has to consider the intercellular network as well.

## Conclusions

EndoNet is, to our knowledge, a unique information resource dealing with the endocrine network. Information about single components of the intercellular network also can be found in other resources like UniProt or HMRbase. The unique approach is the inclusion of connection events, like a secretion or the binding event of a messenger to a receptor. Comprising this information a network is built that represents the feasible signal flows in the intercellular network. The web interface provides access to these data in different views including basic network processing methods. The user defined networks can be downloaded to allow further studies in system biology or for the integration with other networks.

## Availability and requirements

EndoNet is available at http://endonet.bioinf.med.uni-goettingen.de. The web interface can be used without restrictions.

## Competing interests

The authors declare that they have no competing interests.

## Authors’ contributions

JD designed the further development of the network and the underlying database and supervised the annotators. He implemented the web interface. EW developed the general idea of a database for endocrine pathways, organized funding for the project and gave helpful feedback. JD and EW wrote the manuscript. Both authors read and approved the final manuscript.
